# A case report: Numb Chin Syndrome due to thalamic infarction: a rare case

**DOI:** 10.1186/s12883-019-1525-x

**Published:** 2019-11-29

**Authors:** Florian Rimmele, Henning Maschke, Annette Großmann, Tim P. Jürgens

**Affiliations:** 1 Department of Neurology, Headache Center North-East, University Medical Center Rostock, Gehlsheimer Str. 20, 18147 Rostock, Germany; 2 Department of Radiology, University Medical Center Rostock, Schillingallee 35, 18057 Rostock, Germany

**Keywords:** Numb Chin syndrome, Trigeminal, Thalamic stroke, Cheiro-Oral syndrome, Sensory neuropathy

## Abstract

**Background:**

Numb Chin Syndrome (NCS), which is also characterized as sensory neuropathy of the mental nerve, describes a mostly unilateral numbness of the chin and lower lip. Benign and malignant diseases are known to cause this circumscribed symptom, which can easily be overlooked or misdiagnosed. In this article we present the very rare case of a clinical NCS caused by thalamic lacunar infarction. As a pure sensory stroke it is a rare variant of the Cheiro-Oral Syndrome (COS).

**Case presentation:**

A 63-year-old male patient received an emergency referral to our department after the patient had noticed a feeling of numbness of the left lower lip and chin on the previous day. The neurological examination revealed an approximately 2 × 3 cm area of hypoesthesia in the area of the chin and left lower lip and the cranial MRI an acute ischemia in the right thalamus.

**Conclusions:**

In this case report we introduce a patient who clinically shows an NCS. Various diseases may be responsible for NCS, including malignancies or even central neurological disorders such as multiple sclerosis. A lacunar thalamic ischemia as a cause of NCS is very rare and to our knowledge described in the literature only in the contex of a COS in three cases. We wish to remind the reader, through this case, of the purely descriptive and syndromal character of the NCS and the importance for detecting underlying diseases. Furthermore we give a brief overview of the NCS and causative disorders.

## Background

Numb Chin Syndrome (NCS), which is also characterized as sensory neuropathy of the mental nerve, describes a mostly unilateral numbness of the chin and lower lip. Sensory impairment is not limited to hypoesthesia, but may present as paresthesia, dysesthesia or anesthesia within the receptive field of the terminal branch of the trigeminal nerve’s mandibular division. In most cases, there is no accompanying facial weakness or dysgeusia, and symptoms are predominantly unilateral, with bilateral numbness only described in approximately 15% of cases [1]. The first case descriptions of NCS were published in the nineteenth century by Bell and Vincent with reference to patients with mandibular injuries or retromandibular tumors, although NCS as a fixed term did not emerge before 1963 [2]. NCS is purely descriptive and syndromal, without providing a pathological classification. Most cases, though, are attributed to injuries caused by dental treatments, including mandibular nerve blocks or odontogenic diseases. However, malignant tumors are also described as a cause of NCS; the most frequent primary tumors in the case of NCS caused by metastases have been found to be by breast cancer, followed by lymphomas, prostate cancer and leukemia [3]. Central and peripheral neurological diseases such as Lyme disease [4], temporal arteritis [5], neuropathies or multiple sclerosis [6], which manifest as NCS, have also been described in the literature. This demonstrates how important it is, with regard to possible treatments or prognoses, to thoroughly clarify the causes of this circumscribed symptom, which can easily be overlooked or misjudged by both patients and clinicians. In the following, we report on a case from our emergency department.

## Case presentation

In July 2018, a 63-year-old male patient received an emergency referral to our department from his primary care physician; on the previous day, the patient had noticed a feeling of numbness of the left lower lip and chin while eating his breakfast. The patient compared the sensation to that after being given anesthetic by the dentist. He reported that when getting up that morning, he had already experienced a feeling of uncertainty and a slightly unsteady gait, but that these had completely cleared after a short time. The patient’s medical history revealed Lyme disease with Bannwarth syndrome in 2017, a pulmonary embolism in 2017, a right vestibular neuropathy in 1998, arterial hypertension, nicotine consumption of 80 pack years, as well as a tonsillectomy in 1982. With regard to medications, the patient took 2.5 mg/d of ramipril for the treatment of arterial hypertension. The neurological examination revealed an approximately 2 × 3 cm area of hypoesthesia in the area of the chin and left lower lip. The patient’s sense of taste and smell were intact and the nerve exits were not painful on palpation. Otherwise, the cranial nerve status presented as normal and the muscles were normal in tone and eutrophic without paresis. The muscle reflexes were symmetrical and in the lower half of the normal range. The sensitivity to touch was normal, and coordination was intact. The cranial MRI revealed an acute ischemia in the right thalamus. Over the further course of the inpatient stay, further cerebro- and cardiovascular diagnostics were conducted with Duplex sonography of the brain-supplying arteries, long-term ECG and blood pressure monitoring, laboratory diagnostics, and transesophageal echocardiography. These revealed an increased risk of arteriosclerosis without indications of an embolic etiology of the ischemia. The patient was put on 100 mg of acetylsalicylic acid for cerebrovascular secondary prevention, a cholesterol-lowering treatment with 20 mg of simvastatin was initiated, and the patient was urgently advised to refrain from further nicotine consumption.

## Discussion

**Fig. 1:**
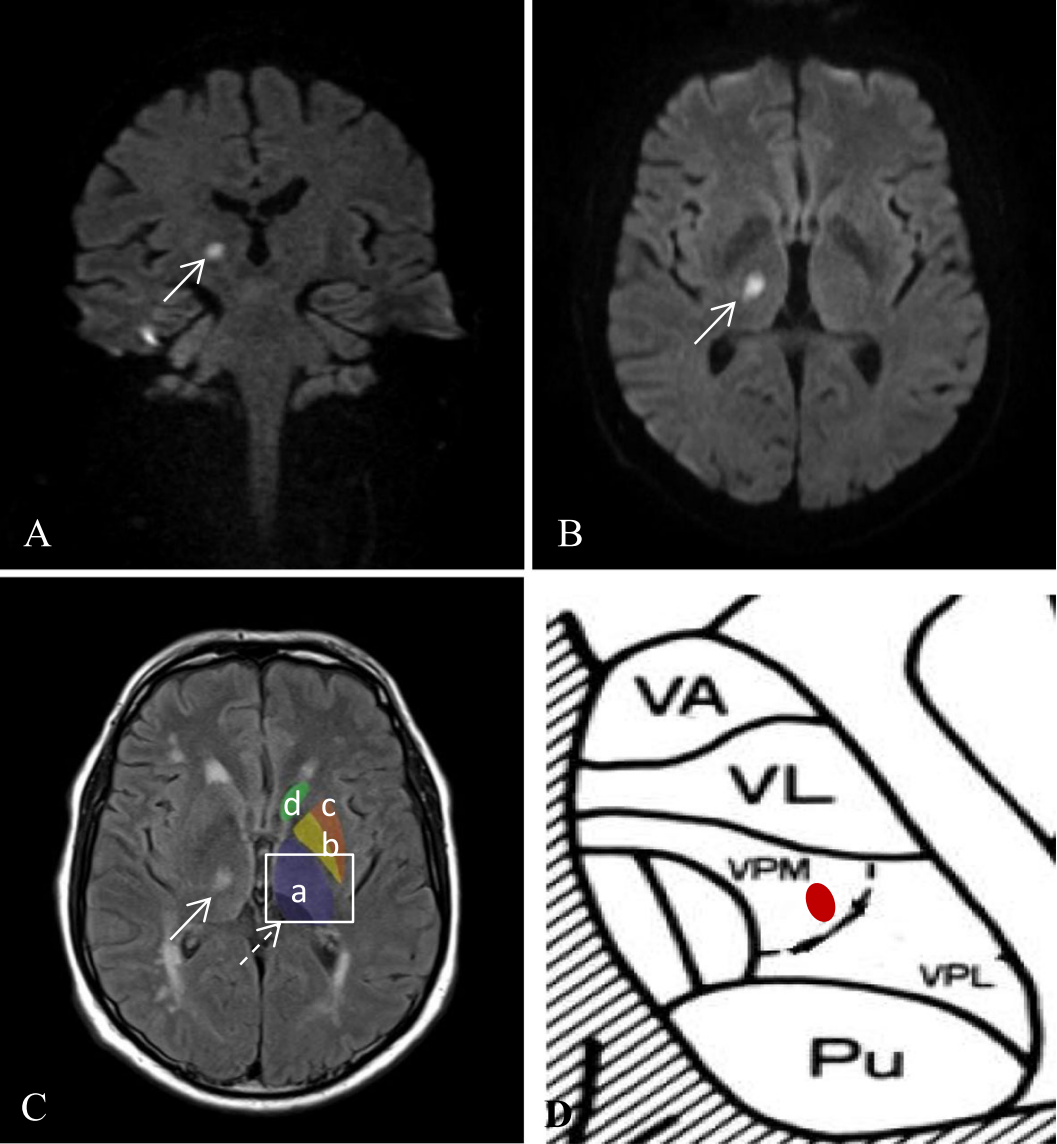
MR images and schematic drawing. **A/B**: Diffusion weighted coronal (**A**) and transversal (**B**) B1000 MRI sequences showing hyperintense signal as a correlate of restricted diffusion in the right thalamus (arrow). **C**: Transversal T2 FLAIR sequence with correlating hyperintense signal within the ischemic lesion of the right thalamus (arrow). Thalamus (a), pulvinar (dotted arrow), globus pallidus (b), putamen (c), caudate nucleus (d). **D**: Thalamus (a) schematic drawing, *Ncl. ventralis* anterior (VA), *Ncl. ventralis* lateralis (VL), pulvinar (Pu), *Ncl. ventralis* posterolateralis (VPL), *Ncl. ventralis* posteromedialis (VPM), hyperintense signal (red); modified after [11]

In the presented case, the leading complaint which brought the patient to the emergency department was the newly emerged feeling of numbness in the area of the left lower lip and the chin. One must be aware that the most diverse of diseases can be causative for the purely syndromal and descriptive NCS. The literature describes dental disorders such as abscesses, root cysts, mental nerve trauma due to ill-fitting dentures, or also lesions of the mandible following fractures, following osteomyelitis, osteonecrosis induced by bisphosphonate treatment or odontogenic infections [2]. Malignancies, in particular mandibular metastases of breast cancer or prostate cancer, can also lead to NCS [3]. The prognosis for patients with NCS due to malignancies is poor, with a mean survival of 6 months or less [7]. However, neurological diseases can also cause NCS: Peripheral-neurological lesions such as diabetic polyneuropathy or chronic inflammatory demyelinating polyneuropathy (CIDP) [8], Lyme’s disease, temporal arteritis [9], but also central-neurological lesions as in multiple sclerosis [10] have all been described as causative. The diverse amount of possible disorders which can clinically manifest as NCS (Table 1), partly involved with a poor prognosis and extensive treatments for the patient, stands in contrast to the initially few deficits in NCS and harbors the danger of trivializing and overlooking it. It is important to view NCS as a “red flag” and to clarify its cause. In the current case, a lacunar ischemia of the right thalamus was found to be the cause of the NCS (Fig. 1). The “pure sensory stroke”, in addition to the “pure motor stroke” and the “dysarthria-clumsy hand syndrome”, is a classic lacunar infarct syndrome. Moreover, the literature describes cases of a pure sensory infarct syndrome, which affects the contralateral finger and the perioral area: the Cheiro-Oral Syndrome (COS), the cause of which is mostly a thalamic lacunar infarction [12]. As a very rare variant of this syndrome, there is an isolated sensory deficit of the face following thalamic lacunar infarction, which to our knowledge has only been published in three cases so far [13–15]. In these three cases, either the whole contralateral side of the face or parts of the distribution territory of the three main trigeminal branches were affected. A circumscribed unilateral numbness corresponding to the distribution area of the mental nerve in the sense of an NCS, as in the case presented here, as a consequence of a thalamic lacunar infarction, has to the best of our knowledge not been previously described in the literature. Topographically, sensory efferents from the upper extremity lie in the ventral posterolateral (VPL) nucleus of the thalamus, while the afferents from the face and the front 2/3 of the tongue lie in the neighboring ventral posteromedial (VPM) nucleus. Lesions of these two core regions lead to COS, which is associated with contralateral hypoaesthesia of the face and fingers [12]. In one case, a lesion at the border between VPL and VPM is described (high signal intensity in T1-weigthed MR imaging), which clinically lead to deafness of the corner of the mouth and the first to third fingers (COS) [11].

**Table 1:** Etiology of Numb Chin Syndrome (NCS)

Nonmalignant Etiologies					Malignant Etiologies	
Dental or iatrogenic Etiologies		Systemic or other Etiologies				
Infectious	Non-infectious	Infectious	Autoimmune	Other	Oropharyngal	Other
Mandibular osteomyelitis Periapical infection Periapical abscess	Mandibular surgery Orthognathic surgery Benign tumor Odontogenic cysts Facial trauma Dental anesthesia Salivary gland biopsy	HIV Syphilis Lyme disease	Chronic inflammatory de-myelinating polyneuropathy (CIDP) Sarcoidosis Multiple sclerosis Systemic lupus erthematosus Giant cell arteritis Primary arteritis nodosa Sjögren’s syndrome	Sickle cell disease Diabetic polyneuropathy Amyloidosis Aneurysms Bisphosponate therapy Mefloquine	Nasopharyngeal cancer Oral cavity/oropharyngeal cancer	Breast cancer Lung cancer Hematological malignancy Lhymphoma Renal tumor Malignant melanoma Gastrointestinal cancer Multiple myeloma Prostate cancer Glioblastoma Medulloblastoma Osteosarcoma Rhabdomyosarcoma Thyroid cancer

In the case presented here, with isolated hypoaesthesia in the area of ​​the left chin and lip, the lesion is located in the area of ​​the right VPM (Fig. 1). The VPM acquires sensory information from the area of ​​the face and lips from fiber tracts of the Lemniscus trigeminalis and the Tractus trigeminothalamicus dorsalis, which are somatotropically arranged within the nucleus. The clinical symptoms vary with infarct size, affected thalamic nuclei and adjacent fiber tracts. The occlusion of a single thalamogeniculate artery results in circumscribed ischemia with an isolated clinical symptom. The isolated symptom, in the case presented here, is most likely attributable to variants of the blood supply through the small penetrating arteries, which emerge from the inferolateral arteries and originate from the posterior cerebral artery [16, 17].

In summary, we present a very rare case, in which a clinical NCS is caused by a thalamic lacunar infarction. Moreover, through this case, we wish to remind the reader of this rare and easily overlooked NCS.

## Data Availability

All data generated or analysed during this study are included in this published article.

## Abbreviations

COS: Cheiro-Oral Syndrome
NCS: Numb Chin Syndrome
VPL: Ventral posterolateral nucleus of the thalamus
VPM: Ventral posteromedial nucleus of the thalamus

## References

[1] Smith RM, Hassan A, Robertson CE (2015). Numb Chin syndrome. Curr Pain Headache Rep.

[2] Assaf AT, Jürgens TP, Benecke AW, Riecke B, Blessmann M, Zrnc TA (2014). Numb chin syndrome: a rare and often overlooked symptom. J Oral Facial Pain Headache.

[3] Galán Gil S, Peñarrocha Diago M, Peñarrocha DM (2008). Malignant mental nerve neuropathy: systematic review. Med Oral Patol Oral Cir Bucal.

[4] Maillefert JF, Dardel P, Piroth C, Tavernier C (1997). Mental nerve neuropathy in Lyme disease. Rev Rhum Engl Ed.

[5] Abilleira S, Bowler JV (2005). The numb chin syndrome as an early manifestation of giant-cell (temporal) arteritis: a case report. Headache.

[6] Oestmann A, Achtnichts L, Kappos L, Gass A, Naegelin Y (2008). "Numb chin syndrome": first presenting syndrome of multiple sclerosis?. Dtsch Med Wochenschr.

[7] Friedrich RE (2010). Mental neuropathy (numb chin syndrome) leading to diagnosis of metastatic mediastinal cancer. Anticancer Res.

[8] Cruccu G, Agostino R, Inghilleri M, Innocenti P, Romaniello A, Manfredi M (1998). Mandibular nerve involvement in diabetic polyneuropathy and chronic inflammatory demyelinating polyneuropathy. Muscle Nerve.

[9] Benito-León J, Simón R, Miera C (1998). Numb chin syndrome as the initial manifestation of HIV infection. Neurology.

[10] Gallud L, Bagan JV, Cervelló A, Jiménez Y, Poveda R, Gavalda C (2006). Multiple sclerosis as first manifestation in oral and facial area: presentation of four cases. Med Oral Patol Oral Cir Bucal.

[11] Shintani S, Tsuruoka S, Shiigai T (2000). Pure sensory stroke caused by a cerebral hemorrhage: clinical-radiologic correlations in seven patients. AJNR Am J Neuroradiol.

[12] Satpute S, Bergquist J, Cole JW (2013). Cheiro-Oral syndrome secondary to thalamic infarction: a case report and literature review. Neurologist.

[13] Arboix A, Tello C, Grivé E, Sánchez M-J (2016). Isolated facial sensory loss due to thalamic lacunar infarction. Acta Neurol Belg.

[14] Chen LL, Youssof S, Karanjia N, Liebeskind DS (2008). Isolated facial sensory loss in stroke restricted to the ventroposteromedial nucleus. Arch Neurol.

[15] Iwasaki Y, Kinoshita M, Ikeda K, Takamiya K, Shiojima T (1991). Oral syndrome: an incomplete form of cheiro-oral syndrome?. Int J Neurosci.

[16] Milisavljević MM, Marinković SV, Gibo H, Puskas LF (1991). The thalamogeniculate perforators of the posterior cerebral artery: the microsurgical anatomy. Neurosurgery.

[17] Herrero M-T, Barcia C, Navarro JM (2002). Functional anatomy of thalamus and basal ganglia. Childs Nerv Syst.

